# Suppurative Otitis Media, Pyæmia

**Published:** 1883-04

**Authors:** Edward Parker Davis

**Affiliations:** Cook County Hospital


### ARTICLE VII.

SUPPURATIVE OTITIS MEDIA, PYÆMIA. A case occurring in Cook County Hospital, in the service of Dr. Henrotin. Reported for the CHICAGO MEDICAL JOURNAL AND EXAMINER, by Dr. EDWARD PARKER DAVIS.

Cases of this character are sufficiently frequent to be familiar to the practitioner; the fatal termination, in this instance, and the opportunity for post mortem examination, attach more than usual interest to the present case. The patient, M. C., female, aged 34, a domestic and native of England, was admitted during the month of February, 1883. She complained of pain in her left ear and the left side of her head, face and neck; deglutition was also very painful. She described her illness as having lasted for five days before admission, during which time she had had chills and profuse perspiration, fever and emesis, with sore throat; her illness began with an ear ache on the left side; some years before, she had an abscess or "boil" in the left ear.

On examination, marked and general icterus is observed, tongue coated dark brown; tenderness in the epigastrium; the liver extending an inch lower than normally. The patient's general nutrition was good; her skin dry and hot; pulse 114, temperature 104.

Chills at various intervals followed the patient's admission, the temperature rising to 105; profuse perspiration, dry tongue, emesis and great debility were observed. Subcrepitant râles were heard at the base of the right lung; pain and tenderness in right hypochondrium. From the time of admission, patient had complained of pain in the left ear, a purulent discharge was noticed, the external meatus was swollen, and difficulty was experienced in gaining access to the middle ear.

Antipyretic and supporting treatment was unsuccessful; the ear was cleansed with carbolized solutions and filled with iodoform, but without avail; the patient dying comatose nine days after admission.

Post mortem examination, as follows:

Seventy-two hours after death, rigor mortis absent; surface of body icteric, a greenish discoloration occupied the posterior triangle of the neck, upon the left side.

Thorax, recent adhesions and ℥ij of serous fluid in the left pleural cavity. Infarctions in both lungs generally disseminated.

Right heart full of dark clotted blood, heart normal, valves competent, abdominal viscera normal, excepting the left kidney, on which the capsule was adherent. Mucous membrane of the bladder covered with pus. In the cranium, between the dura mater and the bones, was found several ounces of pus in the posterior fossa; at the base of the petrous bone was a carious sinus leading to the middle ear which was found to be enclosed by carious walls and filled with sanious pus.

The middle ear was lined with pus and its walls were carious. Making its exit at the jugular foramen, pus had followed the carotid sheath, separating the artery and vein, and passing to the clavicle; a pus sheath of thickened tissue was found exterior to the internal jugular vein.

The mastoid cells were carious and infiltrated with pus.

The cerebrum was not abnormal.

The posterior portion of the left lobe of cerebellum was softened and discolored, apparently from the pressure of the subjacent pus.

For the twelve years ending in '76 eighty-nine cases of neglected otorrhœa which resulted in death, serious exfoliation of bone, or necessitated a severe operation, are cited by a single writer on diseases of the ear (Burnett). Observers of the hospital and dispensary cases are familiar with the appearance of the disease at its earlier stages. The case will suggest to the general practitioner, the importance of an early diagnosis before the suppurative process has become chronic; the operating surgeon will be urged to undertake operative procedure that the advantages of of drainage and disinfectants may be secured before intra-cranial perforation has taken place.

COOK COUNTY HOSPITAL.

